# Dispersal and gene flow in free-living marine nematodes

**DOI:** 10.1186/1742-9994-10-1

**Published:** 2013-01-28

**Authors:** Sofie Derycke, Thierry Backeljau, Tom Moens

**Affiliations:** 1 Department of Biology, Marine Biology section, Ghent University, Krijgslaan 281, S8, 9000, Ghent, Belgium; 2 CeMoFE, Ghent University, K.L. Ledeganckstraat 35, 9000, Ghent, Belgium; 3 Royal Belgian Institute of Natural Sciences (Joint Experimental Molecular Unit), Vautierstraat 29, 1000, Brussels, Belgium; 4 Department of Biology, Evolutionary Biology Group, University of Antwerp, Groenenborgerlaan 171, 2020, Antwerp, Belgium

**Keywords:** Connectivity, Cryptic species, Dispersal, Gene flow, Life history, Marine nematodes, Population genetics, Phylogeography

## Abstract

Dispersal and gene flow determine connectivity among populations, and can be studied through population genetics and phylogeography. We here review the results of such a framework for free-living marine nematodes. Although field experiments have illustrated substantial dispersal in nematodes at ecological time scales, analysis of the genetic diversity illustrated the importance of priority effects, founder effects and genetic bottlenecks for population structuring between patches <1 km apart. In contrast, only little genetic structuring was observed within an estuary (<50 km), indicating that these small scale fluctuations in genetic differentiation are stabilized over deeper time scales through extensive gene flow. Interestingly, nematode species with contrasting life histories (extreme colonizers vs persisters) or with different habitat preferences (algae vs sediment) show similar, low genetic structuring. Finally, historical events have shaped the genetic pattern of marine nematodes and show that gene flow is restricted at large geographical scales. We also discuss the presence of substantial cryptic diversity in marine nematodes, and end with highlighting future important steps to further unravel nematode evolution and diversity.

## Review

Marine nematodes are amongst the most abundant and diverse Metazoa in marine sediments [1,2]. Estimates of species diversity, including terrestrial and parasitic species, vary widely and range from 10^5^[3] to 10^8^[4]. This huge taxonomic diversity encompasses a wide variety of feeding strategies and life history characteristics, but has at the same time hampered ecological studies because species identification is difficult. Consequently, ecological studies on free-living nematodes typically pool species into functional groups based on different feeding strategies [5], tail shape [6], body size [7], life history [8], or a combination of several of these parameters [9]. Next to these ecological studies, considerable work has been done over the last decades to provide an evolutionary framework for the phylum Nematoda [10,11], with a special focus on terrestrial [12], marine [13,14] or parasitic nematodes [15]. These studies show that convergent evolution is a frequent phenomenon for nematode morphology, feeding strategy and habitat. In contrast, there are only few studies on the importance of micro-evolutionary processes (gene flow, genetic drift and selection) for nematode evolution, even for parasitic nematodes [16]. Defining the scales of connectivity among marine populations and identifying the barriers to dispersal and gene flow are however crucial to understand the ecological and evolutionary properties of populations and the dynamics and persistence of populations under environmental changes.

Gene flow describes the exchange of genetic information between populations through migration, whereas dispersal is defined as the movement of individuals from one genetic population to another [17]. Consequently, from a population genetics perspective and for species where individuals rather than eggs or propagules are the mechanism for dispersal, dispersal and gene flow are synonyms [18]. Both terms are used throughout this review. For the marine environment, barriers to gene flow are not always obvious, and factors influencing connectivity among marine populations are roughly divided into physical (e.g. ocean currents, habitat characteristics) and biological (e.g. predation, behaviour) [19]. Retention of organisms in their native area [20] or water currents [21] can strongly limit marine dispersal, which may lead to structured populations [22].

In what follows, we aim to provide a state of the art on dispersal and gene flow at ecological (i.e. one to a few generations) and evolutionary time scales (i.e. hundreds of thousands of generations), and the factors that may influence them (such as life history, habitat and historical events), for marine nematode populations. We also discuss the current knowledge on cryptic marine nematode diversity and end by identifying key questions for future population genetic studies of marine nematodes.

### Dispersal in free-living marine nematodes at ecological time scales

Dispersal is one of the most important life history traits for species evolution and persistence. Dispersal allows organisms to escape unsuitable environmental conditions, avoid competition and increase their distribution range. Dispersal distance is generally correlated with the presence and duration of pelagic larval stages in the water column [23], but there are many exceptions [24], with dispersal being species, season and location specific. Free-living marine nematodes do not have planktonic or pelagic larvae, eggs are generally deposited in situ, and their body size is so small that active dispersal over large distances is likely to be limited. Nevertheless, nematodes are able to actively move in the sediment [25,26], while others can actively emerge into and swim in the water column [27]. Large-bodied nematodes of the family Oncholaimidae rapidly colonize carcasses of fish and macrofauna, probably at least in part by active swimming [28]. They, and other nematodes, may use a variety of chemical cues in their environment to direct their movement towards particular patches [29,30], although it is unclear over what distances such chemotaxis can occur. Some nematode species form non-feeding dauer larvae which are resistant to many environmental stresses [31,32] and which in some species are often found attached to other invertebrates [33]. Such vector associations are known for terrestrial species and may account for dispersal over considerable distances [34], but their role for dispersal of marine nematodes is less documented (a list of commensal marine nematodes in Crustacea is provided by Sudhaus [35]). Passive dispersal of marine nematodes can occur through the ballast water of ships [36], but probably more importantly, through hydrodynamic forces [37]. The presence of nematodes in the water column is largely determined by their vertical distribution and abundance in the sediment [38]. Different nematode genera can also show different vertical distributions in the water column [39] as well as differential abilities to settle back to the sediment [40]. Next to hydrodynamic forces, species-specific traits such as feeding ecology [41,42], behaviour [40], or body morphology [42] influence dispersal ability of marine nematodes. Similar active dispersal abilities have been observed in the deep sea [43-45], but here nematodes are far less abundant in the water column than in shallow-water habitats. The complex interactions between habitat, hydrodynamics and species-specific traits lead to high variation in dispersal patterns through space and time [41,46], which in turn may lead to a high degree of patchiness in nematode community composition [45,47].

**Figure 1:**
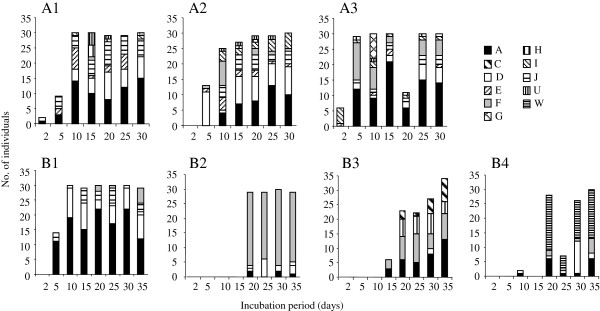
**Genetics of colonizing *Litoditis marina.*** MtDNA haplotype composition of the developing *L. marina* populations during the course of the experiment for each patch separately. **A1A3** refer to the three patches in site A that were incubated amidst permanent *Fucus* stands, while **B1B4** refer to the four patches in site B that were incubated in the absence of permanent algal stands. Distance between algal deposits within a site averaged 2 m, while site A and B were approximately 2 km apart. A-W represent different haplotypes. Note the late colonization and the low number of haplotypes of patches B2-B4, and the dominance of different haplotypes among B1-B4 (Figure from [48]).

Only limited information is available on the effects of dispersal at ecological time scales on population genetic structure in free-living marine nematodes. *Litoditis marina* typically frequents decaying and standing macroalgae in the intertidal, which form a rather transient habitat with frequent local extinctions when algae are decomposed. In such temporal habitats, the ability to disperse enables them to survive the strong fluctuations in habitat availability. *L. marina* produces dauer larvae, and due to its high reproductive capacity and short generation time it is an efficient colonizer that can establish populations from one or a few gravid females. To investigate the effect of colonization dynamics of *L. marina* on neutral genetic variation within and among patches in close proximity (≤ 1 km) to each other, Derycke et al. [48] performed a field experiment in which the genetic diversity of *L. marina* on newly colonized algae was surveyed during one month at two contrasting sites in an intertidal salt marsh: in one site, defaunated algae were incubated amongst permanent algal stands that can act as a source population, while no algal stands were present in the second site [48]. Algal deposits near the permanent algal stands were more rapidly colonized, reached a fivefold higher density of nematodes and had a higher genetic diversity than algal deposits ca 1 km away from the source population (Figure 1). Nevertheless, *L. marina* colonized these distant patches within 10 days, showing that dispersal of this nematode at this scale is substantial. In these distant patches, mtDNA haplotypes that were rare in the source population of the permanent algal stands were abundant suggesting that founder effects and genetic bottlenecks structured these populations (Figure 1). Hence, dispersal at ecological time scales clearly influences the genetic variation within and between patches. Since these colonization dynamics are likely to be related to the ephemeral nature of the habitat and the high reproductive output of *L. marina*, knowledge on the biology and ecology of nematode species is crucial to correctly interpret population genetic data and make conclusions on gene flow and population connectivity.

### Dispersal in free-living marine nematodes at evolutionary time scales

Dispersal in the marine environment can be studied by capture-recapture studies [20] or by determining geochemical signatures in calcified structures (otoliths, shells), but these techniques cannot be applied to minute invertebrates lacking calcified structures. Therefore, dispersal has frequently been estimated indirectly from mathematical modeling or genetic data. The genetic structure is the result of the simultaneous action of evolutionary processes (gene flow, genetic drift and selection, Table 1) across thousands of generations, whereas field studies are restricted to ecological time scales spanning a limited number of generations.

**Table 1:** Evolutionary processes leading to an increased or decreased genetic differentiation between natural populations of species

**Evolutionary process**	**Genetic differentiation between populations**	**Genome wide effects**
Mutation	Increases	No
Gene flow	Decreases	Yes
Genetic drift	Increases	Yes
Divergent selection	Increases	No
Balancing selection	Decreases	No

Gene flow and genetic drift act on the whole genome, while mutation and selection are acting on specific genomic regions.

Population genetic studies typically look at allele frequencies which are used to calculate F_st_ values [49,50] and related statistics, to infer to what degree genetic drift has driven groups of individuals towards fixation of alternative alleles. Therefore, these statistics are suitable to infer genetic structure caused by genetic drift, which is very often correlated with dispersal estimates [51], but processes other than gene flow may also be responsible for this structuring [52]. It is, for example, possible to have perfectly isolated populations between which F_st_ can be comparatively low, simply because both populations are not fixed for alternative alleles [53-55]. Especially when highly variable markers such as microsatellite loci are used, additional statistics such as D or F'_st_ can more adequately reveal genetic differentiation between populations [56,57]. Obviously, one should not blindly look at F_st_ values to infer gene flow, but also explore the raw data (e.g. whether alleles are shared or not between populations). Next to these theoretical aspects, the genetic structure of marine species can be influenced by a variety of biological (e.g. life-history [58-60]) and physical (e.g. water currents [61]), as well as by the interplay between biological and physical factors [62]. In what follows, we review the effects of life history, habitat characteristics and long-term history on the population genetic structure of marine nematodes measured by Φ_st_ (which is similar to F_st_, but also takes sequence divergence into account, whereas F_st_ is based on allele frequencies only [63]).

#### The importance of life history for population genetic structure in marine nematodes

Life histories are known for only a limited number of marine nematode species [e.g. [1,64-66]. Bongers et al. [31] categorized the expected colonizer characteristics of marine nematode genera based on ecological and biological information. This colonizer-persister (cp) scale varies between 1 (extreme colonizers with short generation times and high reproductive output) and 5 (extreme persisters with long generation times and few offspring). Nematode species with cp = 1 are expected to show little genetic structuring because of their ability to rapidly colonize new habitats, while species with cp = 5 are expected to show more pronounced genetic structuring. On the other hand, genetic structuring of marine species with substantial differences in life history and taxonomy can be very similar [67].

**Figure 2:**
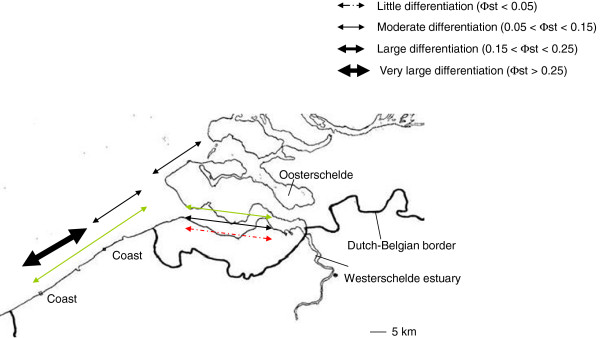
**Genetic structuring in marine nematodes at small geographic scale (<100 km).** The sampling area encompasses the Westerschelde estuary and the Oosterschelde in The Netherlands, and two Belgian coastal locations. The strength of differentiation is based on [53]. Black arrows: *Litoditis marina*; Green arrows: *Halomonhystera disjuncta*; Red arrow: *Bathylaimus assimilis*.

*Litoditis marina* and *Halomonhystera disjuncta* have very short generation times and a high reproductive output [25,68], enabling them to rapidly colonize new habitats [48]. Both species are abundant on decomposing and standing macroalgae in the intertidal, and have a cp value of 1 [31] or 2 for *H. disjuncta* if the presence of a dauer stage is taken as a prerequisite for a cp-score of 1 [69]. Both morphospecies are actually complexes of cryptic species [70,71] and population genetic structure in the most abundant species of the *L. marina* species complex (PmI) within the Westerschelde estuary was low but significant (Φ_st_ = 0.075, p < 0.0001, data recalculated from [70], Figure 2). In view of the ephemeral habitat and strong colonization dynamics of *L. marina*[48], changes in the genetic diversity are likely to occur over time. When sampled in four consecutive seasons, 11% of the genetic variation within each of the five locations in the Westerschelde could be attributed to differences among seasons (Φ_sc_ = 0.11, p < 0.0001, [72]). This confirms that genetic composition of populations changes over time in *L. marina*[72]. Although a significant genetic differentiation was observed among populations within seasons (Φ_sc_ = 0.14, p < 0.0001), there was no significant differentiation among populations when the four seasons were pooled by locations (Φ_ct_ = 0.01, p = 0.262). Since *L. marina* populations are highly unstable over time, this result suggests that the genetic differences caused by extinction-colonization dynamics become unimportant or balance each other when several time points are taken into account. Similar patterns of genetic structure have been observed in the dominant species (GD3) of the *Halomonhystera disjuncta* species complex in the Westerschelde ([71], Figure 2). Four locations were sampled in two different seasons, and a low but significant genetic structuring was observed among locations within seasons (Φ_sc_ = 0.086, p < 0.001, recalculated data from [71]), and temporal differences in genetic differentiation were observed within locations (Φ_sc_ = 0.086, p < 0.001). Again, when the genetic data from the two seasons were pooled within locations, no significant spatial differentiation was observed within the Westerschelde estuary (Φ_ct_ = −0.0042, p = 0.48). Although colonization-extinction dynamics can lead to genetic structuring at small geographical scales, such effects are no longer observed at time scales covering several generations.

**Figure 3:**
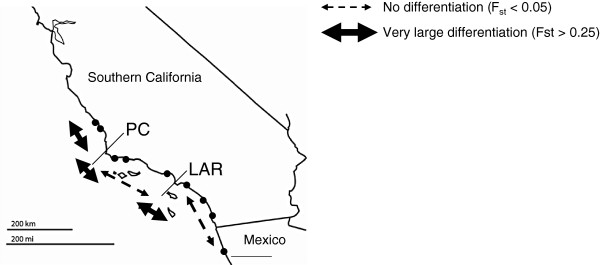
**Genetic structuring in *Thoracostoma trachygaster *at large geographic scale (>500 km).** The study area is situated along the Californian coast, and includes two biogeographic barriers: Point Conception (PC) and the Los Angeles Region (LAR). F_st_ values are based on those reported in [74].

If life-history characteristics are important in determining the genetic structure of nematode populations, pronounced differences in genetic structure would be expected in nematode species with a very long generation time and low number of offspring, because they have much smaller chance of colonizing new habitats than species with short generation times and high reproductive output. The nematode *Thoracostoma trachygaster* lives in holdfasts of kelp along the Californian coast [73], and the genus *Thoracostoma* has a cp value of 5 [31]. Although passive dispersal can still be significant in *T. trachygaster* because of its association with algal holdfasts, intuitively its very long generation time (probably only one or two generations per year) and few offspring would render successful establishment in new habitats less likely compared to *L. marina* and *H. disjuncta*. To exclude effects of well known biogeographic barriers, such as Point Conception and the Los Angeles Region, we investigated the genetic structure of *T. trachygaster* using populations that were continuously distributed along the coast with exclusion of these two barriers, and at a geographical scale of less than 100 km, to be comparable with the geographic scale of the sampling in the Westerschelde (data recalculated from [74]). When taking into account mtDNA allele frequencies and mutations, no genetic structuring was observed within the southern Californian Bight (Φ_st_ = 0.035, p = 0.16; Figure 3). This may suggest that K-strategists do not necessarily have a strong genetic structure. Similar results have been observed for K-strategists in other phyla [75]. At fine geographical scales (< 300 km), shared environmental drivers such as water currents or habitat characteristics may cause similar genetic patterns in species with quite different life histories [67].

#### The importance of habitat characteristics for population genetic structure in marine nematodes

*Bathylaimus assimilis* is an endobenthic nematode species that in contrast to *L. marina, H. disjuncta* and *T. trachygaster,* is not associated with macroalgae. Dispersal through rafting is therefore unlikely for *B. assimilis*. Moreover, several endobenthic nematode species show vertical migration in the sediment [76], and possibly avoid in this way erosion and resuspension in the water column. Therefore the passive dispersal potential of *B. assimilis* is expected to be lower than that of *L. marina* and *H. disjuncta*. Because *B. assimilis* is a less efficient colonizer than *L. marina* and *H. disjuncta*, and because it has an endobenthic life style, its population genetic subdivision in the Westerschelde is expected to be more pronounced than that of *L. marina* and *H. disjuncta*. Yet, a COI sequence analysis of 173 specimens from four locations in the Westerschelde suggested only a weak, but still significant structuring (Φ_st_ = 0.044, p < 0.0001; Figure 2). Although *B. assimilis* lives in the sediment, it can occasionally be observed in the water column [42], increasing its potential for passive dispersal. In conclusion, at small geographical scales of 50 km, population genetic structuring does not seem to depend on whether a nematode is an epiphytic or endobenthic species (but see section on suggestions for future research).

Although gene flow in marine nematodes seems to be quite substantial at scales of 50 km, adding two nearby coastal locations to the Westerschelde data generated Φ_st_ values that were an order of magnitude larger than the values obtained for the Westerschelde populations alone (*Litoditis marina* PmI: Φ_st_ = 0.12 – 0.28, p < 0.0001, data recalculated from [72]; *Halomonhystera disjuncta* GD3: Φ_st_ = 0.11 – 0.13, p < 0.0001 [71]; Figure 2). The stepping stone model assumes that dispersal declines with geographic distance, resulting in an increase in genetic dissimilarity between populations that are more distant from each other [77]. Such isolation by distance (IBD) is supported when there is a positive correlation between genetic and geographic distance [78]. Since there are no obvious barriers to gene flow between the Westerschelde and coastal locations, the more pronounced genetic structuring observed may be caused by geographic distance. However, no significant correlation was observed between genetic and geographic distances for the most widespread species of the *H. disjuncta* complex [71]. For *L. marina*, IBD was found in only one season [72]. In several cases, significant pairwise genetic differentiation was observed between close populations, while no significant differentiation was observed between distant populations. This ‘chaotic genetic patchiness’ pattern is quite common for the marine environment [67], and can be explained by the nonlinear movement of organisms due to turbulent and nonlinear water currents. Taking into account water currents [61] and other environmental data [67] is therefore essential to interpret population genetic data and connectivity in the marine environment. Next to the estuarine and coastal locations, *L. marina* was also sampled in the Oosterschelde, a semi-estuarine environment that is partly closed from the sea by a storm surge barrier, which may provide a higher level of isolation. The habitat type (coastal, estuarine or semi-estuarine) had an impact on the genetic patterns observed within *L. marina* (PmI): 11.15% of the variation could be explained by habitat type (Φ_ct_ = 0.11, p < 0.0001), but a comparable amount of variation (13.09%) was observed by differences between populations within each habitat type (Φ_sc_ = 0.14, p < 0.0001, data recalculated from [72]. As shown for marine invertebrates with larval dispersal stages [61,67,79,80], these results indicate that water currents and ecological characteristics of the habitat may be equally important drivers for the genetic structure of marine nematodes than geographic distance or life history characteristics alone.

#### The importance of geological history for population genetic structure in marine nematodes

Quite a number of marine nematode species show a widespread geographic distribution [81-83], indicating that long distance dispersal can also occur. Next to life history and habitat, historical events such as land mass drift, sea level rises and glacial cycles have influenced the current distribution and population genetic structuring of many marine invertebrates [84]. For the North Atlantic, the quaternary glacial cycles have had dramatic impacts on species distributions, with many species being forced to migrate to the south during glacial periods, followed by recolonization of the northern areas during interglacial periods. These distributional changes have left a genetic imprint, with northern populations being genetically less diverse, and southern populations being genetically richer [85]. Phylogeographical studies in the marine environment have also pinpointed refugial areas e.g. [84], recolonization routes and genetic breaks in a variety of marine organisms. Such genetic breaks can ultimately lead to speciation.

**Figure 4:**
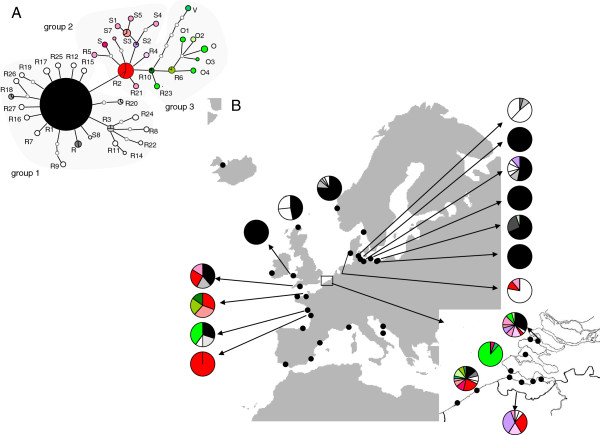
**Distribution of haplotypes and haplotype groupings in the NE Atlantic of *Litoditis marina *(PmII). A**. Relationships among mtDNA haplotypes based on statistical parsimony network. Haplotypes are divided into three groups, corresponding to their geographic distribution and relationships. Haplotypes of group 1 are coloured in black-grey-white, haplotypes of group 2 in red-pink-purple and haplotypes of group 3 in green. Haplotypes found in one location are coloured white, pink or dark green, haplotypes that are shared between locations have a shaded grey, pink or green colour. **B**. Distribution of haplotypes in the NE Atlantic. Group 1 haplotypes are continuously distributed from the Bay of Biscay over the English Channel and the British Isles into the North Sea and the Baltic Sea. Second haplotype group co-occurred with group 1, but was absent from the Baltic Sea and the most northern locations in the NE Atlantic. Most haplotypes of group 3 were restricted to the English Channel and the North Sea (Figure from [81]).

All species of the *L. marina* species complex sampled on a pan European scale showed strong genetic structuring (Table three in [81]), demonstrating that gene flow at larger geographical scales is restricted. When integrating historical processes that have shaped the distribution of temperate species in the North Atlantic with the observed genetic patterns in the *L. marina* complex, the evolutionary history of the species complex becomes visible. For the two most widespread species of the complex (PmI, PmII), a genetic break along the British Isles was observed, and two possible postglacial recolonization routes of northern areas were suggested, one around the British Isles, and one through the English Channel [81] (Figure 4). The Southern Bight of the North Sea acted as a secondary contact zone between these routes. These results illustrate that the quaternary ice ages have influenced the genetic pattern of marine nematodes. Moreover, several population pairwise Φ_st_ values were non-significant, despite populations being separated by obvious geographical barriers (e.g. Baltic and Mediterranean). This illustrates again the chaotic genetic patchiness and suggests that additional ecological factors are influencing the genetic structure of marine nematodes.

Next to glacial cycles, well known biogeographical barriers often coincide with genetic breaks between populations on either side of the barrier [86,87]. *Thoracostoma trachygaster* was sampled along the Californian coast [74], where Point Conception (PC) [88] and Los Angeles Region (LAR) [59] are well-known biogeographic barriers. PC is associated with genetic breaks in high dispersal species, while LAR coincides with genetic breaks in low dispersal species [59,89]. *T. trachygaster* showed a strong genetic structuring along the Californian coastline (Φ_st_ = 0.28, p < 0.001), with a significant amount of this variation being explained by differences between populations north and south of PC and, within the Southern Californian Bight, between populations north and south of LAR (Figure 3, [74]).

Clearly, these studies illustrate that historical processes and biogeographic barriers have strongly affected the genetic variation of marine nematode populations. Furthermore, since these historical events are still detected in the present day genetic composition of marine nematodes, gene flow in marine nematodes must be restricted at such large geographical scales.

### ‘Collateral’ outcomes of population genetic studies: cryptic species

Population genetic and phylogeographic studies typically investigate a large number of specimens from several populations across a species’ geographic range. The majority of known species are however based on descriptions of small numbers of specimens from single or just few localities [90], thereby ignoring the extent of natural variation. Furthermore, the widespread distribution of nematodes is in contrast with their limited dispersal abilities at large geographic scales. This so-called “meiofauna-paradox” [91] may be explained by the presence of cryptic species within what were previously thought to be generalist species [92]. The term “cryptic species” refers to taxa that are morphologically similar, but that belong to different gene pools. Such cryptic diversity occurs in a variety of metazoan taxa and biogeographical regions [93], and may be particularly abundant in the marine environment [94]. This may be because many marine species rely on chemical cues for mate choice and gamete recognition [22,95,96], as well as for ecological interactions [97]. Chemotaxis is used by free-living nematodes to detect food sources [98,99] and parasitic nematodes are able to detect conspecifics in hosts [100]. It is therefore likely that taste and smell are also important for mate recognition in marine nematodes, but no data are currently available to confirm this.

Morphological similarity can be the result of strong divergent selection on non-visual mating signals [101], or, alternatively, morphological stasis may be the result of ecological constraints, where adaptive evolution favors similar phenotypes over and over again [102]. Whatever the speciation process, leaving cryptic diversity unrecognized will bias the interpretation of biogeographical and ecological patterns [101].

Nematode morphology is generally thought to be conserved, leading to speculations on the presence of substantial cryptic diversity in this taxon [101]. However, in a meta-analysis of animal cryptic diversity, cryptic species were not more common in nematodes than in other metazoan taxa [93]. Our population and phylogeographic studies of marine nematodes have revealed the presence of cryptic diversity in various degrees: 10 cryptic species were found in *Litoditis marina*[103], five in *Halomonhystera disjuncta*[71] and three in *Thoracostoma trachygaster*[74,104]. These different numbers of cryptic species may be explained by different sampling efforts, with *L. marina* having been sampled most intensively during four seasons [72] and at a paneuropean scale [81]. When looking at just one season and at the scale of a single estuary (the Westerschelde), two to three cryptic species were found in *L. marina* and *H. disjuncta*. These numbers are quite high considering a geographical scale of less than 50 km, and one might wonder whether this is typical for fast-growing, opportunistic species with rapid reproduction and high numbers of offspring. Preliminary results for the endobenthic monhysterid *Theristus acer* in the Westerschelde show three deeply divergent clades in the COI gene (Derycke, unpublished data). Assuming that these lineages represent cryptic species, the presence of cryptic species seems therefore not to be correlated with life history traits. Instead, the prevalence of cryptic diversity in species with different life histories and from different areas suggests that it is a common phenomenon for marine nematodes. Nevertheless, we did not find any indications of cryptic species in the endobenthic enoplid *Bathylaimus assimilis* within the Westerschelde (Derycke, unpublished data), so caution is needed when making such generalizations based on the limited data available.

Species in species complexes were delimited using the phylogenetic species concept with reciprocal monophyly of nuclear and mitochondrial gene trees. For nematodes, this approach is well-suited [105]. Subsequent detailed morphological studies have shown that the cryptic taxa in these complexes do differ in morphometric characteristics [103,104,106]. However, such differences in morphometry may at least partly be related to environmental conditions such as food availability and temperature, and are thus less suited to delineate or describe species. Detailed morphological studies may however also find diagnostic characters between cryptic species [104,107]. In this way, genetic studies can pinpoint groups that deserve closer morphological study, and can greatly enhance taxonomic studies in small organisms lacking easily observable morphological characters.

Despite the substantial increase of the discovery of cryptic species over the last decade [101,108], only little autecological information is available for cryptic species. For estuarine invertebrates, cryptic species can, for instance, show different tolerances to salinity which can explain their partly overlapping distribution ranges [92,109]. The field distribution of the cryptic nematode species shows that several species tend to co-occur [70,71,74,81], and that temporal fluctuations in species abundances are pronounced [71,72]. Furthermore, at a paneuropean geographical scale, several cryptic species seem to have restricted distributions [81], which may point to differential ecological tolerances/preferences for abiotic factors. Laboratory experiments have shown that two of the four cryptic *L. marina* species (PmI and PmIII) from the Westerschelde show a faster population development at a salinity of 15 psu than at a salinity of 25 psu [110]. Furthermore, when the four species of *L. marina* were combined in a multi species treatment, interspecific interactions reduced the population development of species PmII and even led to the total extinction of species PmIV. These interspecific interactions were also clearly affected by salinity, suggesting that fluctuations in abiotic factors may at least in part drive the coexistence of cryptic nematode species at local scales [110].

### Where to go from here?

#### Nematode population genetics with multiple markers

The population genetic data of marine nematodes are exclusively based on COI, the usefulness of which has been well-documented [87,111]. Although mtDNA is usually treated as if it evolves in a neutral manner, recent studies suggest that selection may also be acting on the mtDNA [112]. Therefore, using independently evolving loci will enhance the correct interpretation of the processes responsible for the observed genetic patterns. Microsatellite loci (see e.g. [113,114] for a background) have become tremendously popular for population genetic studies because of their high intraspecific variability, which allows investigation of contemporary gene flow at small geographical scales. Yet, although microsatellite loci have been used in population genetic studies of parasitic nematodes [115-117] and in the model nematode *Caenorhabditis elegans*[118,119], no such data are currently available for marine nematodes.

#### Understanding the role of ecology in nematode population genetics

Life history, morphology, behavior and habitat-associated traits all contribute to dispersal ability, but hitherto their relative importance for the genetic structure in marine nematodes remains largely unknown. Comparing the genetic structure between several species differing in one of these traits can contribute to unravel the relative importance of these traits for micro-evolutionary processes [51]. For instance, by including additional nematode species isolated from the same geographic area but with life histories that differ from that of efficient colonizers such as *L. marina* and *H. disjuncta,* the effects of life history on population structure and nematode evolution can be explored. Similarly, including species with different habitat preferences should highlight habitat related effects on genetic structure. Since both *L. marina* and *H. disjuncta* occur on macroalgae, they are likely to have substantial passive dispersal capacity, and their dispersal ability may thus be much larger than that of typically endobenthic nematode species. Although for the time being this is not confirmed by our data on *Bathylaimus assimilis,* one would expect that if an endobenthic life style restricts dispersal in marine nematodes, then one should find higher Φ_st_ values in these species.

#### Unravelling the importance of environmental drivers for nematode dispersal through sea scape genetics

Next to species-specific ecological characteristics, dispersal in marine nematodes may be driven by environmental parameters. The genetic structuring in marine nematodes so far does not seem to correlate with geographic distance, but instead shows chaotic genetic patchiness: population pairwise F_st_ values are often significant between populations in close proximity, while they are not for populations that are further apart. This pattern may well be caused by the hydrodynamic currents in the study area, as well as by other ecological factors. Coupling hydrodynamic [61,120] and other environmental data [67] with genetic structuring, the so-called sea scape genetics approach [121], can help to sort out the causes of spatial patterns in marine population genetics.

#### Investigating the importance of selection for population differentiation in nematodes

Next to the analysis of neutral genetic variation, understanding the importance of selection on genetic structuring is essential for predicting how populations will respond to changing environments and to understand evolutionary diversification. Neutral loci across the genome will be similarly affected by demography and the evolutionary history of populations, while loci under selection will often behave differently and therefore reveal ‘outlier’ patterns of variation [122]. Next generation sequencing (NGS) makes it more feasible than ever to identify genes underpinning adaptive evolution in non-model organisms. Single Nucleotide Polymorphisms (SNP’s) are very common and distributed across the genome, and can be screened for many individuals from different populations through Restriction site Associated DNA sequencing (RADSeq) [123,124]. RADseq tags digested DNA from a large number of individuals, which are then pooled and sequenced using Illumina. The resulting sequence reads can be analysed without a reference genome by aggregating identical reads into unique sequences. Subsequently, unique sequences with only a small number of mismatches are clustered, and SNP’s can be scored between alleles at the same locus [125]. In this way, RADSeq generates thousands of genetic markers in a large number of specimens at a reasonable cost [125]. By choosing populations living in e.g. different temperature or salinity conditions, which are amongst the most important forces for local adaptation in marine invertebrates [126], and comparing the genetic differentiation between these populations at different genomic regions, it becomes possible to pinpoint those genomic regions that are under selection.

#### Investigating the influence of genome evolution on population genetic patterns in marine nematodes

The advance in sequencing technology has generated an unprecedented amount of genome and transcriptome data from, mainly parasitic, nematode species [127,128]. Horizontal gene transfer is a common phenomenon in plant parasitic nematodes [129,130], but has not been reported in free-living nematodes [130]. Comparative genomics between the free-living *Caenorhabditis elegans* and *C. briggsae* have revealed extensive intrachromosomal rearrangements, but remarkable conservation of chromosome organization and synteny [131]. Comparison of mitochondrial genomes across parasitic nematode species has revealed a large number of gene rearrangements, large duplications, and the use of both DNA strands to encode genes [132]. Furthermore, the presence of minicircular [133] and multipartite mitochondrial genomes has been documented [134]. The effect of these genomic differences on population genetic patterns and nematode dispersal is unknown. Clearly, genome sequences of marine nematodes are urgently needed to investigate the prevalence of these phenomena in marine nematodes and their effect on interpreting population genetic patterns.

## Conclusion

Experimental field studies have demonstrated that dispersal of marine nematodes at ecological time scales (i.e. < 10 generations) is substantial in the estuarine environment. The use of genetic data revealed that colonization dynamics strongly affect the genetic composition of local patches, with founder effects and bottlenecks causing strong differentiation among nearby patches. At deeper time scales, these genetic differences seem to disappear and populations become homogeneous. Consequently, gene flow in the marine nematodes analysed so far is substantial at geographical scales of 50 km, but is strongly restricted at larger geographical scales (several 100’s of kilometers). This scale is tentative, and depends on a variety of environmental factors. Our data suggest that life history (short generation time and high reproductive output vs. long generation time and low reproductive output) and habitat preference (algae vs. sediment) may be less important drivers for dispersal in marine nematodes, but additional analyses of the genetic structure in other nematode species are required to confirm these observations.

## Competing interests

The authors declare that they have no competing interests.

## Authors’ contributions

SD conceived and wrote the manuscript, and analysed the data. TB and TM critically reviewed the manuscript and improved its intellectual content. All authors read and approved the final manuscript.
